# Rurality and race in heart failure risk: Insights from the Southern Community Cohort Study

**DOI:** 10.21542/gcsp.2024.4

**Published:** 2024-01-03

**Authors:** Susy Kotit

**Affiliations:** Aswan Heart Centre (AHC), Aswan, Egypt

## Abstract

Introduction: Rural–urban health disparities are apparent in the burden of disease and health outcomes, including cardiovascular disease (CVD), specifically heart failure (HF). However, the factors influencing these disparities are not fully understood.

Study and results: Among 27,115 participants in the Southern Community Cohort Study (SCCS) (mean age: 54 years (47-65)), 18,647 (68.8%) were black, 8,468 (32.3%) were white, and 20% resided in rural areas. Over a median 13-year follow-up period, 7,542 HF events occurred (rural = 1,865 vs. urban = 5,677). The age-adjusted HF incidence was 29.6 (95% CI, 28.9–30.5) and 36.5 (95% CI, 34.9–38.3) per 1,000 person-years for urban and rural participants, respectively (*P* < .001). The risk of HF associated with rurality varied by race and sex. Rural black men had the highest risk across all groups (HR, 1.34; 95% CI, 1.19–1.51) (age-adjusted incidence rate: 40.4/1000 person-years (95% CI, 36.8–44.3)) followed by black women (HR, 1.18; 95% CI, 1.08–1.28) and white women (HR, 1.22; 95% CI, 1.07–1.39). Rurality was not associated with HF risk among white men (HR, 0.97; 95% CI, 0.81–1.16).

Lessons learned: This large study shows that rural populations have an increased incidence of HF, which is particularly striking among women and black men, independent of individual-level biological, behavioral, and sociocultural risk factors. It also shows the need for further investigation into the rurality-associated risk of HF, the impact of preventive care utilization on the risk of HF and interpersonal, community, or societal factors that could contribute to rural–urban disparities. This will help to guide public health efforts aimed at HF prevention among rural populations.

## Introduction

Rural–urban health disparities are apparent in the burden of disease and health outcomes^1^ in cardiovascular disease (CVD)^2^, specifically in heart failure (HF). Over 6.5 million adults in the US have HF^3^, with over 1 million new diagnoses each year. The projected increase in prevalence by 2030 is approximately 46%, affecting up to 1 in 33 individuals^4^.

Heart failure is associated with considerable morbidity and mortality. Despite advancements in treatment, the 1-year mortality rate remains high at 30%, increasing to approximately 40% at 5 years^5^.

**Figure 1.**
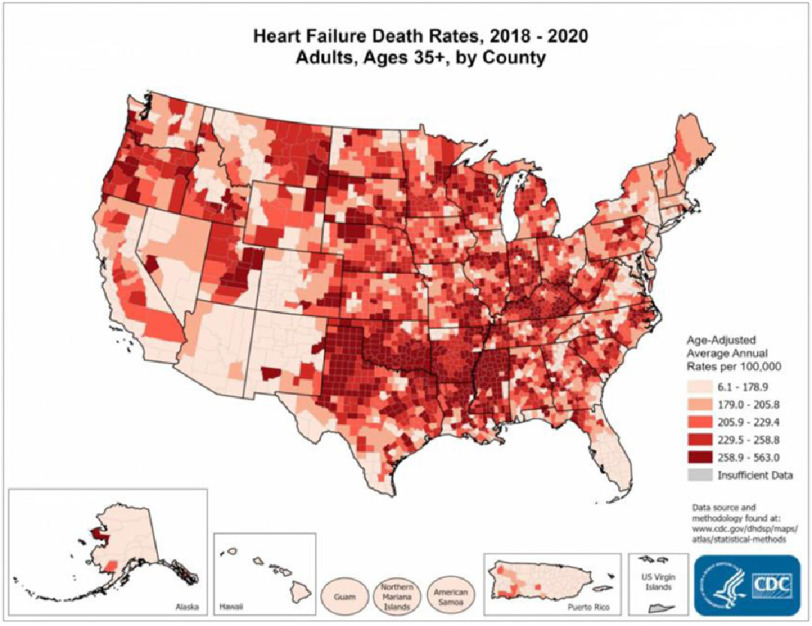
Heart failure mortality across the US. The map shows that concentrations of counties with the highest heart disease death rates –meaning the top quintile –  are located primarily in Mississippi, Louisiana, Arkansas, Oklahoma, Texas, Kentucky, Tennessee, Indiana, Illinois, and Wisconsin. Pockets of high-rate counties also were found in Oregon, Utah, Montana, South Dakota, and Nebraska^9^.

There is substantial variability in the prevalence of HF and mortality varies geographically (Figure 1). Specifically, rural populations in the United States experience a disproportionate HF mortality burden, compounded by racial inequities ^6,7^. The black population, particularly in the rural South, is at a higher risk of dying from HF than those in urban areas^7^. Black men residing in rural areas are at a higher risk of dying from heart failure (HF) than black men in urban areas and white men in both rural and urban areas. However, the factors influencing these disparities are not fully understood, and whether they are due to a higher incidence of HF among the black population in rural areas or due to societal factors remains unclear ^8^.

Understanding the association between living in rural areas and an increased incidence of heart failure, as well as the discrepancy in heart failure risk, with gender and race serving as compounding issues, is essential to address geographic-, gender-, and race-based disparities and develop varied preventive approaches across individuals to reduce those gaps.

### The study

The Southern Community Cohort Study (SCCS) was a population-based cohort of low-income, underserved participants^10,11^ from 12 states across the south-eastern United States^12^. The prospective cohort study analyzed data of 27,115 African American, Black, or non-Hispanic White participants without HF at enrolment between 2002 and 2009, who received care *via* community health centers for underserved populations^11,13^ and followed them up until 2016.

The SCCS examined the incidence of HF by rurality status across race and sex in a large community cohort of black and white adults with the objective of analyzing the incidence as well as the sex and race variability of HF among rural adults, and determining if rurality independently contributes to HF risk beyond CV risk factors and socioeconomic status (SES). Rurality was defined by Rural-Urban Commuting Area codes at the census-tract level for participant addresses^14–18^.

**Figure 2.**
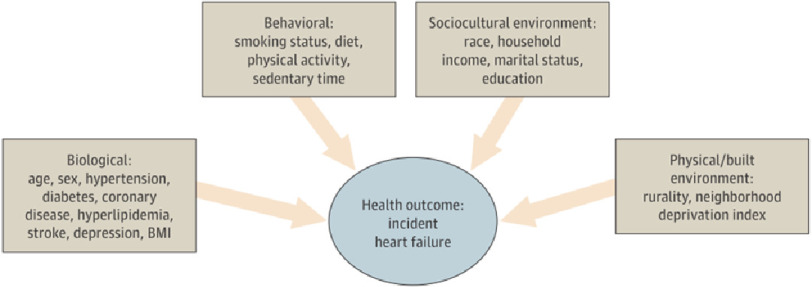
Application of the National Institute of Minority Health and Health Disparities (NIMHD) Research Framework^24^. BMI, body mass index. Permission to use the panel A diagram provided by the National Institute on Minority Health and Health Disparities.

Data on demographic characteristics and medical history were collected, including age, sex, race, annual household income, education, marital status, hypertension, diabetes, coronary disease, hyperlipidemia, stroke, depression, body mass index (BMI), smoking status, and insurance coverage (Figure 2).

Diet was assigned a score according to the Healthy Eating Index (HEI), ranging from 0 to 100 based on alignment with the 2010 US Dietary Guidelines^19,20^. Physical activity was quantified as standard metabolic-equivalent hours per day (calculated from the sum

of light, moderate, and vigorous activities)^21,22^ and total hours spent sitting per day^22^. Neighbourhood deprivation index (NDI) was determined by a composite score within four categories (education, employment, occupation, poverty), previously derived in the SCCS *via* principal components analysis^12,15,23^.

Incident and prevalent HF were ascertained using diagnosis codes from the International Classification of Diseases, Ninth Revision (ICD-9) and International Statistical Classification of Diseases and Related Health Problems, Tenth Revision^11^. Incident HF was defined as the first-ever occurrence of an HF code documented in Medicare institutional or outpatient claims. The incidence of HF was calculated by person-years of follow-up and age standardized. Sequentially adjusted Cox proportional hazard regression models were used to test the association between rurality and incident HF.

Mortality was used as a censoring variable and was ascertained through the Social Security Administration’s vital status service and the National Death Index^11^.

## Results

Among 27,115 participants, the median (IQR) age was 54 years (47-65), 18,647 (68.8%) were black, and 8,468 (32.3%) were white; 5,556 participants (20%) resided in rural areas. Individuals in rural areas were older, more commonly female, had a slightly higher BMI and rates of hypertension, diabetes, coronary disease, and hyperlipidemia; similar rates of stroke; and a lower rate of depression. Diet and exercise were similar between urban and rural participants; however, rural participants were more likely to be married, less educated, and less likely to be current smokers. Urban participants were slightly more likely to live in neighborhoods with a higher level of deprivation, but overall income did not differ by rurality.

Over a median 13-year follow-up period, 7,542 incident HF events occurred (1,865 among rural and 5,677 among urban participants). The age-adjusted HF incidence was 29.6 (95% CI, 28.9–30.5) per 1000 person-years for urban participants and 36.5 (95% CI, 34.9–38.3) per 1000 person-years for rural participants (*p* < 0.001). Black men, black women, and white women living in rural areas had higher age-adjusted HF incidence rates than their respective urban counterparts.

The risk of HF associated with rurality varied by race-gender group, as Rural Black men had the highest risk of HF across all groups in every sequential model, including the final model (HR, 1.34; 95% CI, 1.19–1.51), with an age-adjusted incidence rate of 40.4 (95% CI, 36.8–44.3) per 1,000 person-years. Black women (HR, 1.18; 95% CI, 1.08–1.28) and white women (HR, 1.22; 95% CI, 1.07–1.39) had a similar increased risk of HF attributable to rurality. In contrast, rurality was not associated with HF risk among white men (HR, 0.97; 95% CI [0.81–1.16]).

After adjustment for demographics (age, gender, race) rurality was associated with a 21% greater risk for incident HF (HR, 1.21; 95% CI, 1.15–1.28), which persisted after adjustment for biological factors, with rural residents experiencing 17% greater risk for incident HF (HR, 1.17; 95% CI, 1.11–1.24). In the final model, rurality was independently associated with a 19% greater risk of HF (HR, 1.19; 95% CI, 1.13–1.26). The association between rurality and HF was not attenuated by adding behavioral risk factors, further addition of sociocultural, racial and ethnic environment covariates, NDI, enrollment source, or insurance coverage.

## Discussion

This study represents the first large population-based study examining the incidence of HF by rurality in the United States, comparing the rates of new-onset HF among rural and urban residents in 12 southeastern states during both inpatient and outpatient HF visits.

The study population reflected the demographics of rural America, namely, older age, lower levels of education, and a greater burden of CV disease and comorbidities^2^. The results revealed rural–urban health inequalities in heart failure risk, with gender and race serving as compounding issues. In this large cohort of predominantly low-income black and white individuals in the southeastern United States, the HF incidence was higher for both white and black women in rural areas, with rural black men experiencing the highest rate of incidence of HF, which is markedly higher than both the HF incidence among urban black men and the previously reported overall HF incidence among black men in SCCS^11^. Importantly, the study found no association between heart failure risk and urban or rural residency for white men.

The substantial excess risk of HF associated with rurality persisted after adjusting for biological, behavioral, and sociocultural risk factors, suggesting that the effect of rurality is not simply a consequence of higher CV risk among rural residents. Furthermore, the association between rurality and incident HF seemed independent of sociocultural factors, including education, income, marital status, and overall neighborhood social environment.

These observations suggest that, beyond individual-level risk indicators and socioeconomic status, the excess risk of HF associated with rurality is driven by societal, community, and interpersonal factors^24^.

Access to health care is believed to be a significant societal driver of rural–urban disparities through inequities in levels of preventive care, with risk factor management being singled out as a top priority for improvement^25^, specifically after HF diagnosis, as rural patients have a longer delay in care, negatively influencing health outcomes.

Barriers to care that contribute to rural–urban disparities include social deprivation, the lack of a landline or cell signal to ensure provision and follow-up of care, longer response times for emergency medical services, longer wait times for specialists, insufficient availability of hospitals in rural areas, distance to health care facilities, barriers to transportation, inequities in access to medical care, and a domain that includes both care-seeking behaviors and logistical access to care.

Inequities in preventive care and risk management also affect disease incidence. Therefore, the association between rurality and incident HF is not merely a manifestation of neighborhood deprivation, but rather suggests that the place-based effect of a rural environment uniquely contributes to risk for HF beyond the Neighborhood Deprivation Index (NDI) and shows the importance of domains beyond the individual level^24^ to understand the real impact of rurality.

Nonetheless, the observations in the study emphasize the importance of considering other domains beyond the individual level^24^ to understand how rurality differentially affects black and white men. One such domain is structural racism, which creates racial inequities through multiple mechanisms such as neighborhood segregation, and discrimination within the health care system, influencing access to the resources necessary for proper heart health such as access to healthy foods and recreation, adequate income and housing, and preventive health care^26–31^. Misogyny is another important domain, as women are often not believed when they complain of illness and their poor health is dismissed and downplayed, affecting the speed of care.

The study included more than 27 000 participants across an entire region; however, most participants had low socioeconomic status (SES), and the majority were identified as black, typically underrepresented in epidemiologic cohorts. One of the important limitations of the study is the fact that most participants were older, white/black, male/female, with low incomes, and covered by CMS, and it is unclear if these findings might be affected by a higher income, private insurance, younger population, and different racial, ethnic, and gender identities in other US regions^13^. Therefore, the findings pertain to the south-eastern United States and may not be generalizable to individuals with a higher income, private insurance and living in other regions.

Furthermore, the study was limited to individual-level domains of influence^24^ and did not look into the reasons behind the geographic and racial health disparities, although some influencing factors, such as limited access to resources, structural racism in racial disparities, healthcare access, and food insecurity.

Although prevalent coronary heart disease was adjusted in the analysis, incident coronary heart disease was not included and may have occurred prior to the onset of HF. Adjustment for process-related measures such as healthcare utilization and longitudinal risk factor control (blood pressure medication and control, statin use, lipid levels, and hemoglobin A 1c control) was not possible, which may explain the observed differences, as rural populations have a greater prevalence of smoking, obesity, physical inactivity, diabetes, hypertension, hyperlipidemia, stroke, and coronary heart disease^2,32–36^.

The results of the Southern Community Cohort Study demonstrate substantial variability in susceptibility to heart failure with an association between living in rural areas and an increased incidence of heart failure and highlight race- and sex-based inequities in heart failure risk. This indicates the importance of examining the effect of rurality by race-sex group, as both white and black women had an increased risk of HF associated with rurality, in contrast to white men, who had no rurality-associated risk of HF.

Important implications for the primary prevention of HF include a need to focus on community or contextual factors that may preferentially impact women or black men living in rural areas, to elucidate the mechanism by which rurality is associated with HF risk, as well as the need for personalized prevention^37^, focusing on rural women and rural black men as key groups.

The findings of this study emphasize the need for a comprehensive approach to address the rural–urban disparities in heart failure risk, addressing societal, community, and interpersonal factors, improving access to care, and promoting personalized prevention strategies, as the exact reasons behind these rural–urban health disparities remain unclear and understudied.

Studies analyzing interventions to prevent heart failure in rural populations to address geographic-, gender-, and race-based disparities, as well as exploring the value of targeted and tailored preventive care in rural populations, are warranted to help develop interventions to correct the rural–urban disparities revealed in this study.

Translating the observational findings of this study into targeted interventions to prevent heart failure, particularly among individuals who bear a disproportionate risk burden, is an important future prospect. Furthermore, increasing access to care and improving the quality of healthcare in rural America and worldwide are paramount.

### Lessons learned

This large study shows that rural populations have an increased incidence of HF, which is particularly striking among women and black men, independent of individual-level biological, behavioral, and sociocultural risk factors, and shows the need for further investigation into the rurality-associated risk of HF to guide public health efforts aimed at HF prevention among rural populations.

Future research should examine the impact of preventive care utilization on the risk of HF and chronic disease among rural residents, as well as the effects of race and rurality on disease incidence and outcomes.

The study suggests the importance of personalized prevention focusing on rural women and rural black men and to elucidate the mechanism by which rurality is associated with HF risk.

Further studies should investigate interpersonal, community, or societal factors that could contribute to rural–urban disparities, such as access to care, a domain that includes both care-seeking behaviors and logistical access to care, to understand the exact reasons behind rural–urban health disparities.
